# The Hedgehog Signaling Pathway is Expressed in the Adult Mouse Hypothalamus and Modulated by Fasting

**DOI:** 10.1523/ENEURO.0276-21.2021

**Published:** 2021-09-29

**Authors:** Patrick J. Antonellis, Staci E. Engle, Kathryn M. Brewer, Nicolas F. Berbari

**Affiliations:** 1 Department of Biology, Indiana University-Purdue University Indianapolis, Indianapolis, Indiana 46202; 2 Stark Neurosciences Research Institute, Indiana University School of Medicine, Indianapolis, Indiana 46202-3082; 3 Center for Diabetes and Metabolic Diseases, Indiana University School of Medicine, Indianapolis, Indiana 46202-3082

**Keywords:** feeding behavior, hedgehog signaling, hypothalamus, primary cilia

## Abstract

The hedgehog signaling pathway is best known for its role in developmental patterning of the neural tube and limb bud. More recently, hedgehog signaling has been recognized for its roles in growth of adult tissues and maintenance of progenitor cell niches. However, the role of hedgehog signaling in fully differentiated cells like neurons in the adult brain is less clear. In mammals, coordination of hedgehog pathway activity relies on primary cilia and patients with ciliopathies such as Bardet–Biedl and Alström syndrome exhibit clinical features clearly attributable to errant hedgehog such as polydactyly. However, these ciliopathies also present with features not clearly associated with hedgehog signaling such as hyperphagia-associated obesity. How hedgehog signaling may contribute to feeding behavior is complex and unclear, but cilia are critical for proper energy homeostasis. Here, we provide a detailed analysis of the expression of core components of the hedgehog signaling pathway in the adult mouse hypothalamus with an emphasis on feeding centers. We show that hedgehog pathway genes continue to be expressed in differentiated neurons important for the regulation of feeding behavior. Furthermore, we demonstrate for the first time that pathway activity is regulated at the transcriptional level by fasting. These data suggest that hedgehog signaling is involved in the proper functioning of brain regions that regulate feeding behavior and that hedgehog pathway dysfunction may play a role in the obesity observed in certain ciliopathies.

## Significance Statement

Here we investigate the expression of hedgehog pathway components in the adult mouse hypothalamus. Using dual-labeling *in situ* hybridization, we show that core components of the signaling pathway are expressed in multiple neuronal cell types within the hypothalamic feeding centers. Our findings also support previous findings that astrocytes are responsive to hedgehog signaling, as determined by *Gli1* and *Ptch1* expression. Using quantitative PCR analysis, we show that hypothalamic hedgehog pathway activity is upregulated in response to fasting and that this response is nuclei specific. These data not only provide a more detailed understanding of hedgehog pathway expression in the adult mouse hypothalamus but also provide direct evidence of a novel role for hedgehog signaling in the physiological response to fasting.

## Introduction

Initially described in a *Drosophila* mutant screen, the hedgehog pathway is now classically recognized for its iterative role in the formation and function of several mammalian tissues and organs such as the limb bud, neural tube, and skeleton (Nüsslein-Volhard and Wieschaus, 1980; Goetz and Anderson, 2010). In mammals, the hedgehog pathway is dependent on the primary cilium as an organizing center (Huangfu et al., 2003; Breunig et al., 2008; Willaredt et al., 2008; Gorivodsky et al., 2009; Stottmann et al., 2009). Components of the hedgehog pathway such as patched (PTCH1), smoothened (SMO), and the Gli transcription factors GLI2 and GLI3 dynamically localize to the primary cilia (Corbit et al., 2005; Haycraft et al., 2005; Rohatgi et al., 2007). Upon binding to the ligand sonic hedgehog (SHH), PTCH1 leaves the cilia, allowing for SMO to enter primary cilia. GLI2 is activated into its transcriptional activator (GLI2A) form, while GLI3R formation is inhibited, leading to the expression of Gli target genes, which includes *Ptch1* and *Gli1* (Chen and Struhl, 1996; Marigo and Tabin, 1996; Bai et al., 2004; Fuccillo et al., 2006). Numerous other genes are known to regulate hedgehog signaling in embryonic development, such as *Gpr161*, an orphan G-protein-coupled receptor (GPCR) shown to localize to cilia and negatively regulate pathway activity (Mukhopadhyay et al., 2013).

Beyond embryonic development, cilia and hedgehog signaling continue to play an important role in the postnatal brain for growth and the maintenance of neural progenitors (Machold et al., 2003; Chizhikov et al., 2007; Breunig et al., 2008; Han et al., 2008; Vaillant and Monard, 2009). Primary cilia are found on almost all mammalian cell types, and their dysfunction underlies a class of human genetic disorders known as ciliopathies (Reiter and Leroux, 2017). Ciliopathies present a wide range of clinical symptoms, some of which are associated with genetic defects in hedgehog signaling such as bone, limb patterning, and genitalia malformations (Umehara et al., 2000; Gao et al., 2001; Hellemans et al., 2003; Mujahid et al., 2018; Khan et al., 2019). Furthermore, the ciliopathy Carpenter syndrome [#20100, Online Mendelian Inheritance in Man (OMIM)] results from mutations in Rab23, a negative regulator of hedgehog signaling. Interestingly, obesity is also a core clinical phenotype of certain ciliopathies such as Bardet–Biedl syndrome (#209900, OMIM), Alström syndrome (#203800, OMIM), and Carpenter syndrome (Jenkins et al., 2007; Alessandri et al., 2010; Marshall et al., 2011; Forsythe et al., 2018). Thus, these genetic disorders implicate a potential role for hedgehog signaling in regulation of energy homeostasis in humans.

Ciliopathy mouse models, as well as conditional animal models of cilia loss, have implicated hypothalamic neuronal cilia-mediated feeding behaviors in obesity (Davenport et al., 2007; Siljee et al., 2018; Wang et al., 2019; Rouabhi et al., 2021; Sun et al., 2021). Neuronal primary cilia preferentially localize GPCRs known to be important for the regulation of energy homeostasis and feeding behavior, such as neuropeptide Y (NPY) receptors 2 and 5, melanin-concentrating hormone receptor 1 (MCHR1) and melanocortin-4 receptor (MC4R; Berbari et al., 2008; Loktev and Jackson, 2013; Siljee et al., 2018). However, the impact of ciliary localization on their signaling capabilities is not well understood. We have previously demonstrated in primary mouse hypothalamic neurons *in vitro*, interactions between a ciliary GPCR and hedgehog signaling, suggesting that the hedgehog pathway may modulate GPCR activity at the cilium in differentiated neurons (Bansal et al., 2019). However, the role of hedgehog signaling *in vivo* in the adult hypothalamus is less clear. *In situ* hybridization studies have shown that *Shh*, *Ptch1*, and *Smo* mRNA are expressed in several regions of adult rat brain, including the hypothalamus (Traiffort et al., 1998, 1999, 2001; Banerjee et al., 2005). Here we characterize in greater detail the expression and transcriptional activity of the hedgehog pathway in the feeding centers of the adult hypothalamus *in vivo*. We also demonstrate that hedgehog pathway activity changes based on feeding status and that this response is absent following the onset of obesity, suggesting a role for hedgehog signaling in the modulation of adult behaviors.

## Materials and Methods

### Animals

All procedures were approved by the Institutional Animal Care and Use Committee at Indiana University-Purdue University Indianapolis. Male C57BL/6J mice (stock #000664) were ordered from The Jackson Laboratory, and they were housed on a standard 12 h light/dark cycle and given food and water *ad libitum* except for experiments as described previously. Chow-fed mice were maintained on a standard chow diet consisting of 13% fat, 67% carbohydrate, and 20% protein caloric content (2014 Teklad, Envigo). High-fat diet (HFD)-fed animals were maintained on a diet consisting of 40% fat, 39% carbohydrate, and 21% protein caloric content starting at 6 weeks of age (catalog #TD95217, Envigo).

### *In situ* hybridization

Brains from 8- to 10-week-old male C57BL/6J mice were harvested and fixed as described previously (Engle et al., 2018). Sections were cut at a thickness of 15 μm and mounted directly on slides, then were postfixed with 4% paraformaldehyde for 16 h at 4°C. The detection of transcripts in brain sections was performed using the RNAscope 2.5 HD Duplex Assay (ACD). Tissue pretreatment was performed according to user manual technical note 320534 Rev A. Probe hybridization, counterstaining, and mounting of slides were performed according to user manual #322500-USM Rev A. Slides were assayed using probes to SHH (catalog #314361), SMO (catalog #318411), GLI1 (catalog #311001), PTCH1 (catalog #402811), GPR161 (G-protein-coupled receptor 161; catalog #318111), AGRP (agouti-related peptide; catalog #400711-C2), POMC (proopiomelanocortin; catalog #314081-C2), MC4R (catalog #319181-C2), MCHR1 (catalog #317491-C2), or GFAP (glial fibrillary acidic protein; catalog #313211-C2) transcripts (ACD). Sections were counterstained with hematoxylin, dehydrated, and mounted using VectaMount (Vector Laboratories). Slides with positive control probe (PPIB-C1/POLR2A-C2; ACD catalog #321651) and negative control probe (DapB; ACD catalog #320751) were run with each experiment. At least three animals were analyzed for each group.

### Quantitative real-time PCR

RNA was isolated, cDNA was prepared, and quantitative real-time PCR was performed as described previously (Bansal et al., 2019). Assays-on-Demand Gene Expression Probes (Applied Biosystems) were as follows: Shh Mm00436528_m1; Ptch1 Mm00436026_m1; Smo Mm01162710_m1; Gli1 Mm00494654_m1; and Gpr161: Mm01291057_m1. Ct values were normalized to β-actin, relative expression was calculated by the ΔΔCt method, and fold change was calculated by normalizing relative expression to the proper control.

### Experimental design and statistical analyses

Whole hypothalamus was collected from 35- to 36-week-old C57BL/6 lean and obese animals that were allowed either *ad libitum* access to food or were fasted overnight. Region-specific micropunches were collected from 7- to 8-week-old, lean, C57BL/6 mice using 1.0 mm Militex Biopsy Punch (Electron Microscopy Sciences). There was a minimum of six animals per treatment group. Statistical analysis was performed using two-way ANOVA and corrected for multiple comparisons. Differences were considered significant when *p* < 0.05. Data are presented as the mean ± SEM.

## Results

**Figure 1.**
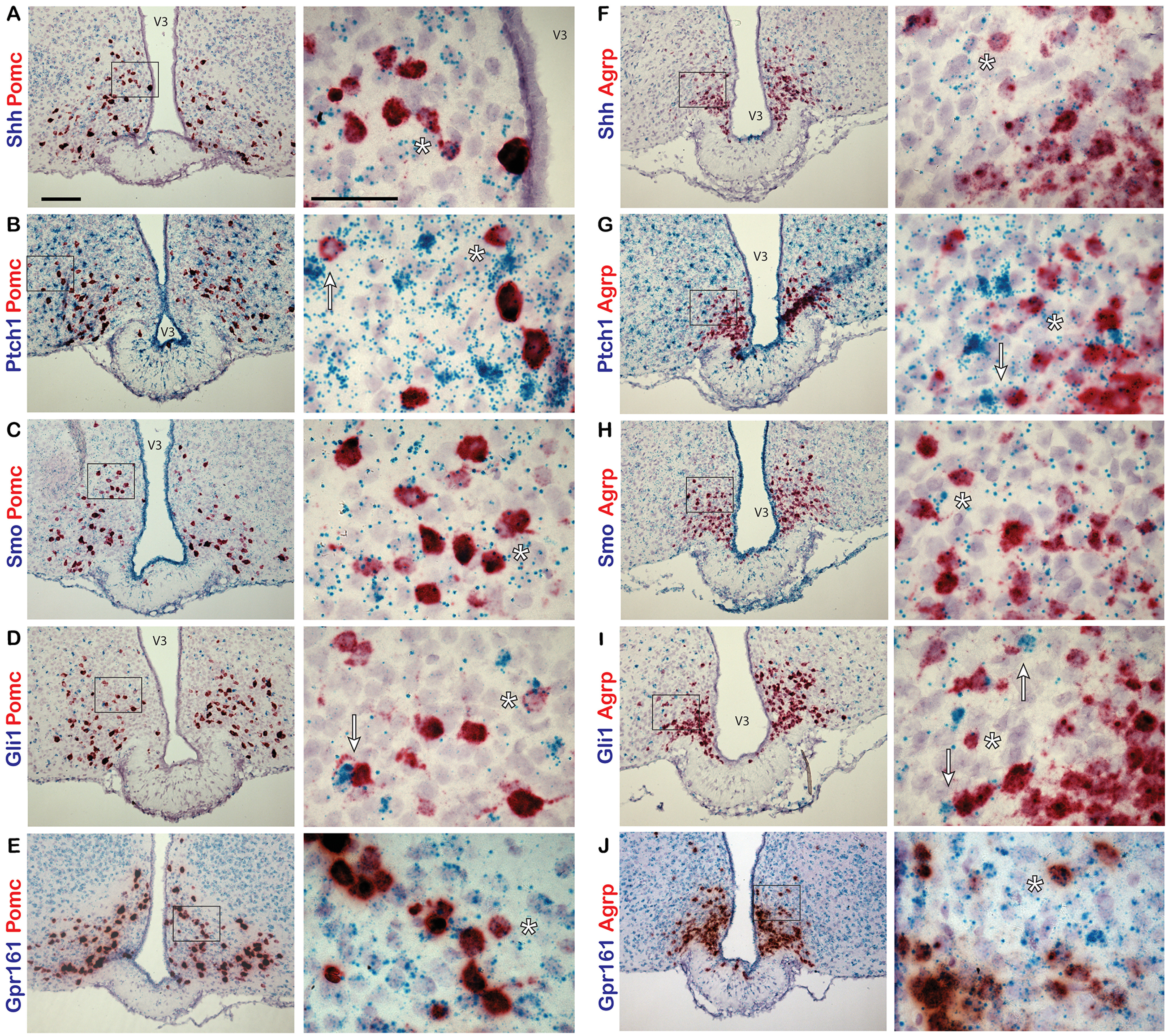
Hedgehog pathway expression in the adult mouse ARC. Dual-probe *in situ* hybridization labeling of the ARC with probes to hedgehog pathway and neuronal gene transcripts. ***A–J***, *Pomc* (***A–E***) and *Agrp* (***F–J***) probes are labeled in red, while *Shh* (***A***, ***F***), *Ptch1* (***B***, ***G***), *Smo* (***C***, ***H***), *Gli1* (***D***, ***I***), and *Gpr161* (***E***, ***J***) probes are labeled in blue. Examples of cells colabeled by both probes are denoted by an asterisk (*). *Pomc*- or *Agrp*-expressing cells adjacent to highly expressing *Ptch1* (***B***, ***G***) or *Gli1* (***D***, ***I***) cells are denoted by an arrow. Right-hand panels are magnified images of the region shown in a black box on the left-hand side. Scale bars: left panels, 100 μm; right panels, 50 μm. V3, Third ventricle.

**Figure 2.**
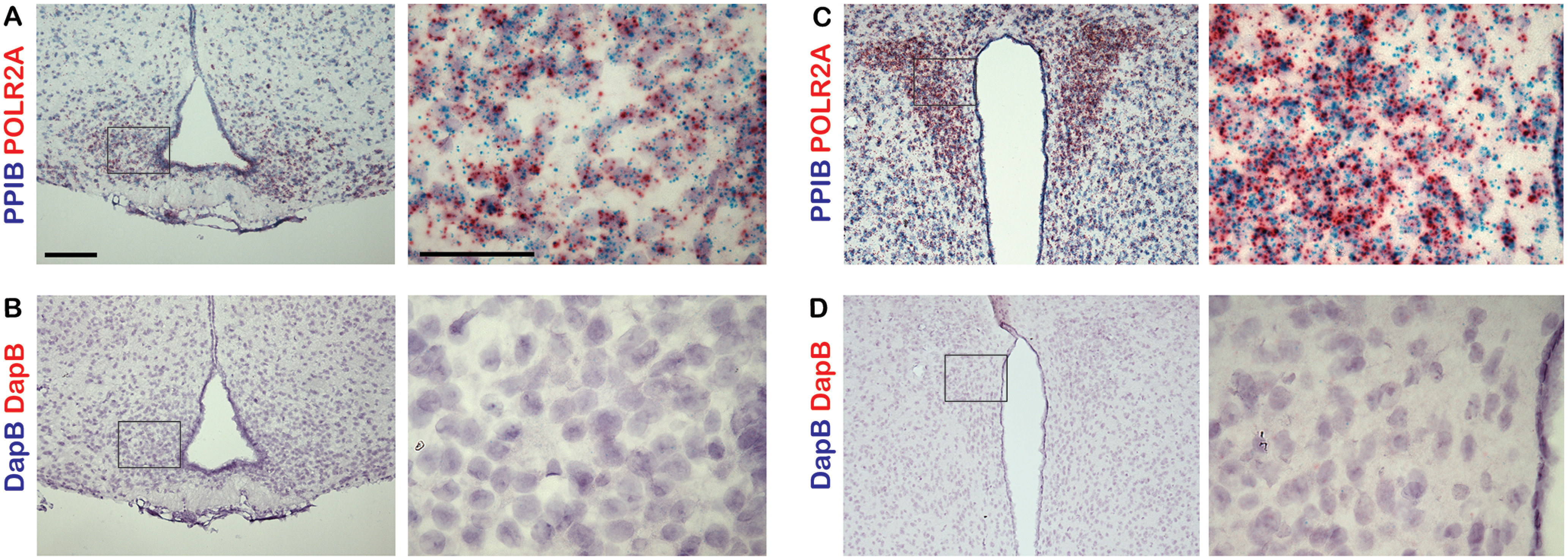
Controls for dual-probe *in situ* hybridization studies. ***A–D***, Sections of adult mouse ARC and PVN were labeled with either positive control probes targeting the mouse genes *Ppib* and *Polr2a* (***A***, ***C***) or negative control probes targeting the bacterial gene *dapB* (***B***, ***D***). Right-hand panels are magnified images of a region shown in a black box on the left-hand side. Scale bars: left panels, 100 μm; right panels, 50 μm.

To determine whether *Shh* and components of the signaling pathway are expressed in the adult mouse hypothalamus, we performed *in situ* hybridization studies. Using a dual-labeling approach, we first assessed whether neurons of one of the feeding centers, the arcuate nucleus of the hypothalamus (ARC), express hedgehog pathway genes. Two major neuronal subtypes within the ARC, the anorexigenic POMC-expressing neurons and orexigenic AGRP/NPY-coexpressing neurons, are crucial for normal energy homeostasis (Belgardt et al., 2009). Hypothalamic sections from adult, C57BL/6 mice were labeled with probes to *Shh*, *Ptch1*, *Smo*, *Gli1*, or *Gpr161* and colabeled with probes to either *Pomc* or *Agrp*. In the ARC, we found that all hedgehog pathway genes assayed were coexpressed in neurons expressing *Pomc* (Fig. 1*A****–****D*) and *Agrp* (Fig. 1*F***–***I*). We also found that *Gpr161* is expressed at a relatively high level throughout the ARC (Fig. 1*E*,*J*). For each experiment, sections were labeled with positive and negative control probes (Fig. 2).

**Figure 3.**
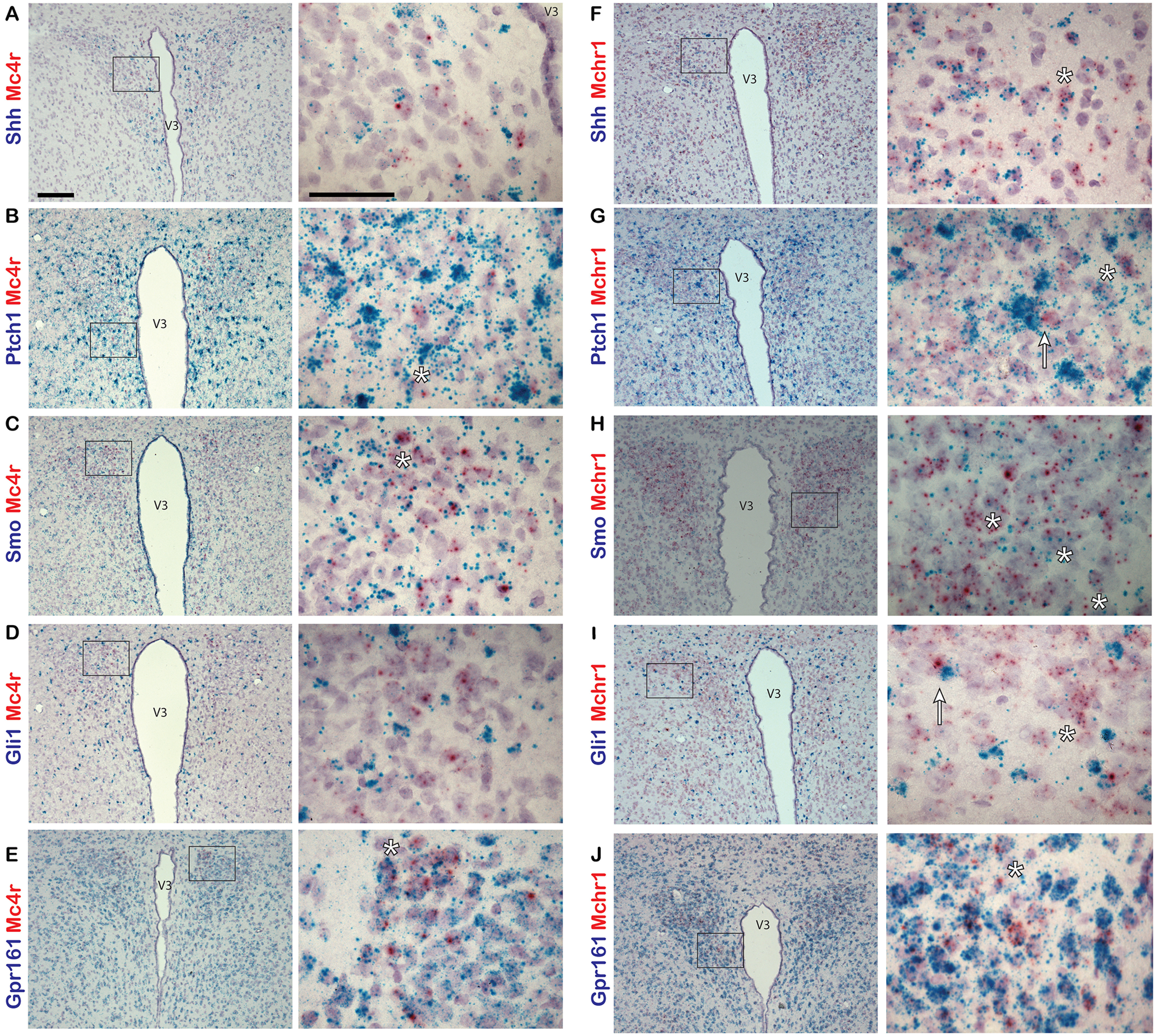
Hedgehog pathway expression in adult mouse PVN. ***A–J***, Dual-probe *in situ* hybridization of probes for hedgehog pathway gene transcripts colabeled with probes to neuronal gene transcripts in the PVN of adult mice. *Mc4r* (***A–E***) and *Mchr1* (***F–J***) probes are labeled in red, while *Shh* (***A***, ***F***), *Ptch1* (***B***, ***G***), *Smo* (***C***, ***H***), *Gli1* (***D***, ***I***), and *Gpr161* (***E***, ***J***) probes are labeled in blue. Example of cells colabeled by both probes are denoted by an asterisk (*). *Mchr1*-expressing cells adjacent to highly expressing *Gli1* cells are denoted by an arrow (***I***). Right-hand panels are magnified images of a region in a black box on the left hand-side. Scale bars: left panels, 100 μm; right panels, 50 μm. V3, Third ventricle.

We next assessed whether *Shh* and its pathway components are also expressed in another feeding center, the paraventricular nucleus of the hypothalamus (PVN). We labeled sections of hypothalamus with probes to *Shh*, *Ptch1*, *Smo*, *Gli1*, or *Gpr161*, and colabeled them with probes to either *Mchr1* or *Mc4r*. Both MCHR1 and MC4R are known to be important for the regulation of energy homeostasis and feeding behavior and are localized to primary cilia in neurons (Berbari et al., 2008; Siljee et al., 2018). In the PVN, we observed relatively few *Mc4r*-positive neurons with sparse incidence of colabeling with probes to hedgehog pathway genes (Fig. 3*A*–*D*). However, *Mchr1*-positive neurons were much more abundant and were frequently colabeled with probes to all hedgehog pathway genes used (Fig. 3*F–I*). As in the ARC, *Gpr161* is expressed abundantly throughout the PVN (Fig. 3*E*,*J*).

**Figure 4.**
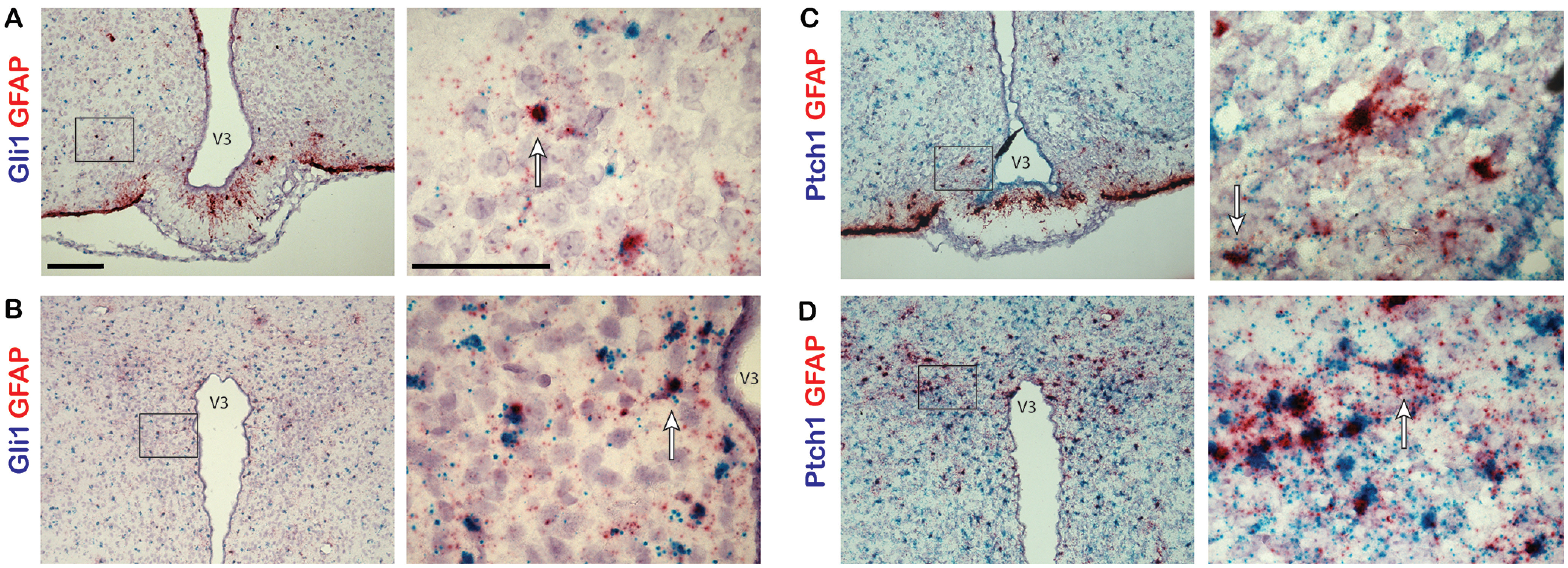
*Gli1* and *Ptch1* expression in astrocytes in adult mouse hypothalamus. Dual-probe *in situ* hybridization was performed with probes for *Gli1* or *Ptch1* and astrocyte marker *Gfap* gene transcripts. ***A–D***, Sections of the ARC (***A***, ***C***) and PVN (***B***, ***D***) from adult mice were colabeled with probes to *Gli1* or *Ptch1* labeled in blue and probes to *Gfap* labeled in red. Example cells that are highly positive for both probes are denoted by an arrow. Right-hand panels are magnified images of a region shown in a black box on the left-hand side. Scale bars: left panels, 100 μm; right panels, 50 μm. V3, Third ventricle.

Interestingly, we observed cells highly positive for either *Gli1* or *Ptch1* expression throughout the ARC and PVN. Some of these cells appeared adjacent to neurons expressing *Pomc*, *Agrp*, or *Mchr1* (Figs. 1*B*,*D*,*G*,*I*, 3*I*, arrows). Previously, it has been reported that astrocytes of the adult mouse brain are responsive to hedgehog signaling and increase *Gli1* expression on pathway activation (Garcia et al., 2010). Therefore, we sought to determine whether these hedgehog-responsive cells were astrocytes by colabeling with probes to either *Gli1* or *Ptch1*, and *Gfap*, an astrocyte marker. In the ARC (Fig. 4*A*,*C*) and PVN (Fig. 4*B*,*D*), cells highly expressing *Gfap* were observed colabeled with *Gli1* or *Ptch1*, suggesting that some of the hedgehog-responsive cells adjacent to neurons may be astrocytes in the hypothalamus.

**Figure 5.**
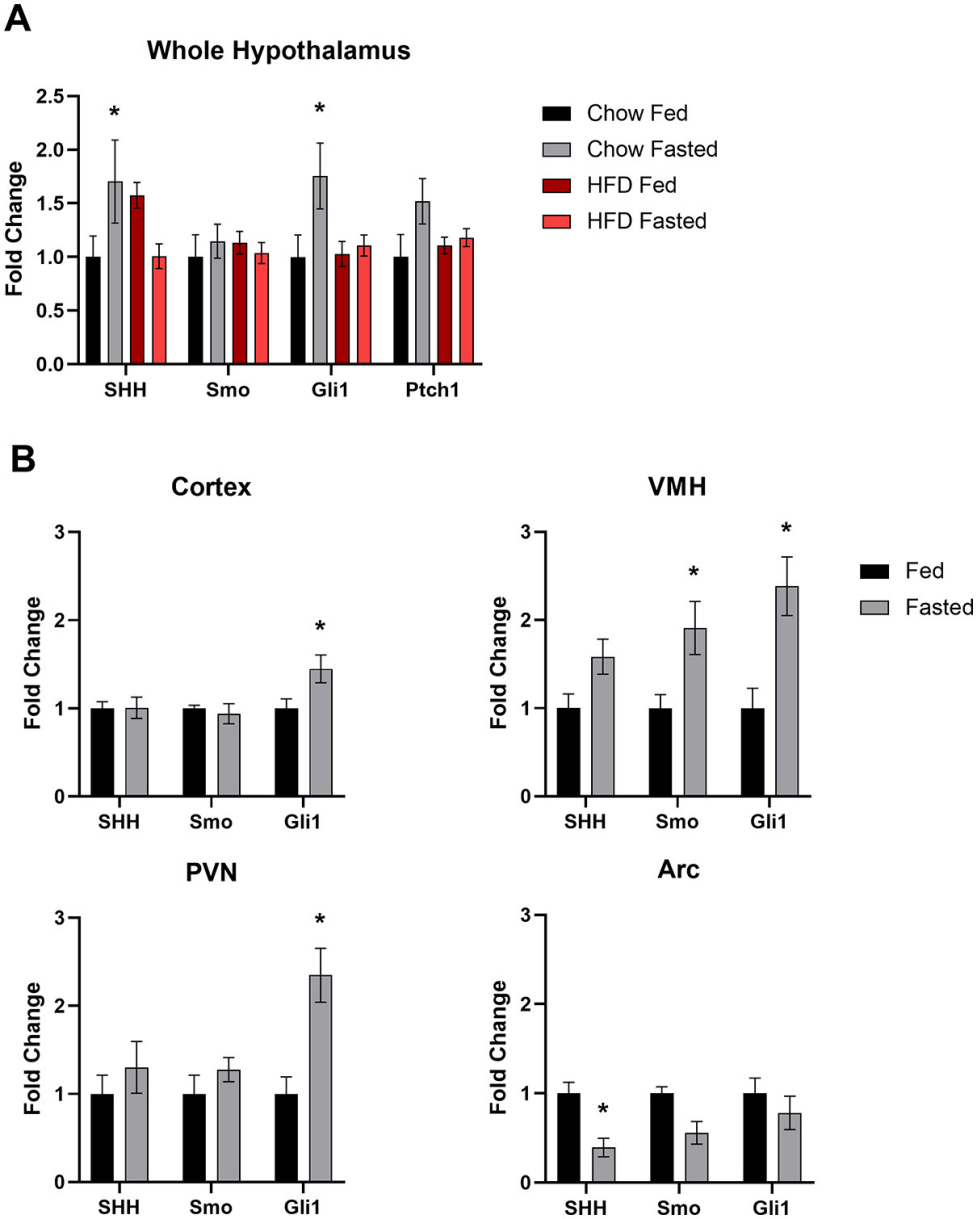
Feeding status mediated changes in hypothalamic hedgehog pathway gene expression. ***A***, Hedgehog pathway gene expression in the whole hypothalamus of adult mice. Lean animals fed a standard chow diet or obese animals fed a high-fat diet were allowed *ad libitum* access to food or fasted overnight. A minimum of six animals were used per treatment group. Whole hypothalamic RNA was used for qPCR. ***B***, Hedgehog pathway gene expression in brain micropunches of adult mice. A total of 16 animals, 8 per treatment group, fed a chow diet were allowed *ad libitum* access to food or fasted overnight. Micropunches were taken from specific nuclei of the hypothalamus and cortex for qPCR analysis. *p < 0.05.

**Figure 6.**
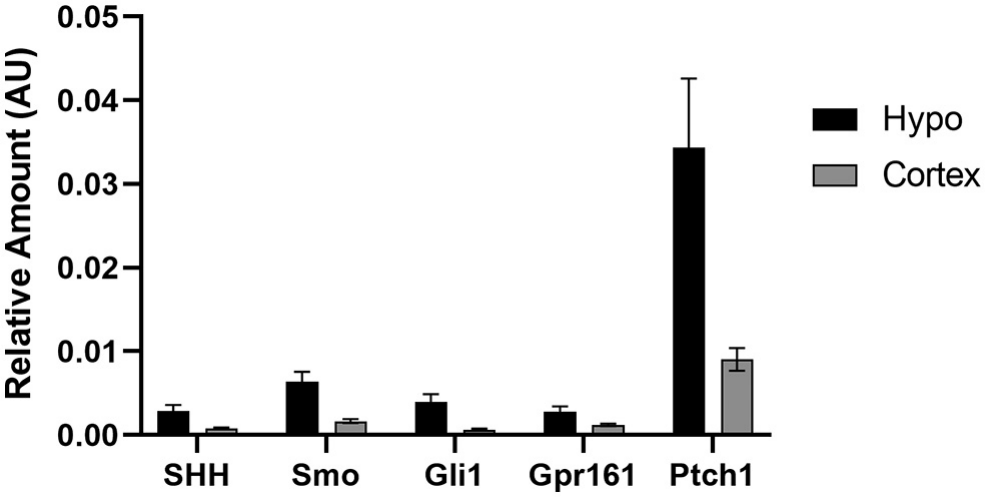
Expression of selected hedgehog pathway transcripts in the adult mouse brain. Whole hypothalamus and cortex were collected for qPCR analysis from six adult animals allowed *ad libitum* access to food.

Since we determined that genes of the hedgehog pathway are expressed in neurons of the adult hypothalamus, we next wanted to determine whether these genes are transcriptionally regulated by physiological changes associated with the normal function of this brain region, such as nutritional status. This was accomplished by gene expression analysis of the whole hypothalamus from chow-fed lean and HFD-fed obese animals in the fed and fasted state. HFD-fed animals weighed significantly more than their chow-fed counterparts at 35 weeks of age (57.1 ± 0.97 vs 34.8 ± 0.75 g; mean ± SEM; *p* < 0.05, Student’s *t* test). Lean and obese animals were allowed either *ad libitum* access to food or were fasted overnight, then whole hypothalamic RNA was collected for quantitative PCR (qRT-PCR) analysis. In lean animals, there was a significant increase in both *Shh* and *Gli1* expression in the hypothalamus after an overnight fast (Fig. 5*A*). The expression of *Ptch1* also was elevated in fasted compared with fed animals, but this effect was not significant (Fig. 5*A*). Strikingly, there were no significant changes in gene expression in the hypothalamus of obese fasted versus fed animals (Fig. 5*A*). Since transcriptional regulation was observed in response to fasting in control chow-fed animals at the level of the whole hypothalamus, we wanted to assess which specific nuclei were contributing to this effect. Once again, control, normal weight C57BL/6J animals were allowed either *ad libitum* access to food or were fasted overnight. Micropunches were then collected from the cortex, ventromedial hypothalamus (VMH), PVN, and ARC for qPCR analysis. We found that fasting that induces an increase in *Gli1* expression in the whole hypothalamus is driven by increases in *Gli1* expression specifically in the VMH and PVN but not in the ARC (Fig. 5*B*). We also found that *Gli1* was upregulated, to a lesser extent, in the cortex (Fig. 5*B*). Additionally, expression of *Smo* was increased in the VMH, while *Shh* gene expression was reduced in the ARC following an overnight fast (Fig. 5*B*). Finally, we performed qPCR on whole hypothalamus and cortex, a brain region known to exhibit hedgehog pathway activity (Garcia et al., 2010; Harwell et al., 2012; Hill et al., 2019), collected from adult animals. We found that all hedgehog pathway genes measured were more highly expressed in the hypothalamus than the cortex (Fig. 6). Overall, these data demonstrate that not only does expression of the hedgehog pathway continue into adulthood in the hypothalamus, a region critical for energy homeostasis, but that specific nuclei respond with transcriptional changes based on feeding status.

## Discussion

Primary cilia are crucial for mediating hedgehog signaling in mammals and, furthermore, certain ciliopathies are associated with pediatric obesity (Engle et al., 2021). Therefore, we focused our efforts on evaluating the expression of the hedgehog pathway in hypothalamic feeding centers of adult mice. Because reliable commercial antibodies for many pathway proteins are currently unavailable, making immunolabelling studies difficult, we used a dual-labeling *in situ* hybridization approach that allowed for sensitive detection of hedgehog pathway gene transcripts in specific adult neurons. Our *in situ* data revealed the broad expression of *Shh* and core pathway components throughout the hypothalamus of adult mice. We found that mRNA for *Shh*, *Ptch1*, *Smo*, *Gli1*, and *Gpr161* could be detected in both the ARC and PVN. Within the ARC, each one of these transcripts are detected in neurons coexpressing *Pomc* or *Agrp*. While a similar labeling pattern was observed in the PVN, transcripts for hedgehog pathway genes were more readily found colabeled with probes to *Mchr1* than *Mc4r*. However, this could potentially be because of the relatively low abundance of MC4R-expressing neurons or low mRNA expression for this particular GPCR. Our results expand on prior studies that found hedgehog pathway expression in the adult rat brain (Traiffort et al., 1998, 1999; Banerjee et al., 2005) by demonstrating that neurons in two nuclei of the hypothalamus important for regulation of feeding behavior express *Shh* and members of its signaling pathway. Somewhat surprisingly, we also found that *Gpr161* is expressed abundantly throughout both the ARC and PVN. This is contrary to previously published findings that used digoxigenin probes *for in situ* analysis of the adult mouse brain and showed a more restricted expression pattern of *Gpr161* in the nucleus accumbens and amygdala with low hypothalamic expression (Ehrlich et al., 2018). Analysis of the amygdala and accumbens was outside the scope of the present study, and relative expression of *Gpr161* between these brain regions was not determined. However, a thorough understanding of this GPCR-negative regulator of hedgehog signaling in the adult brain may reveal themes for its roles in cilia-mediated behaviors.

Interestingly, in both the ARC and PVN we observed cells with high expression of either *Ptch1* or *Gli1* immediately adjacent to neurons expressing *Pomc*, *Agrp*, or *Mchr1*. By colabeling sections of hypothalamus with probes to *Gfap* and *Gli1* or *Ptch1*, we were able to identify some of these hedgehog-responsive cells as *Gfap*-positive astrocytes. While neurons of the adult mouse hypothalamus express *Shh* and components required for its signal transduction, the cells that are most responsive to hedgehog signaling, as indicated by high levels of *Gli1* and *Ptch1* expression, may in fact be astrocytes. This supports previous findings showing that not only do neurons produce *Shh*, but subpopulations of mature astrocytes in the forebrain are responsive to hedgehog signaling (Garcia et al., 2010). It remains unclear why these *Gfap*-positive cells outside of known neurogenic niches exhibit high levels of *Gli1* and *Ptch1* relative to neighboring neurons.

There is growing evidence suggesting that the hedgehog pathway is involved in the regulation of whole-body energy homeostasis. It has been demonstrated in both the fat body of *Drosophila* and the adipose tissue of mice that hedgehog signaling regulates adipocyte differentiation (Suh et al., 2006; Pospisilik et al., 2010). Circulating forms of Hedgehog have been detected, and during *Drosophila* larval development have been shown to be secreted from the gut and to act on multiple tissues to coordinate development with nutrient availability (Palm et al., 2013; Rodenfels et al., 2014). Given these findings, we also analyzed the expression of *Shh*, *Smo*, and *Gli1* in the adult mouse hypothalamus in response to changes in metabolic state and nutritional status. We compared transcriptional regulation of the hedgehog pathway in both lean control diet-fed animals and obese HFD-fed animals. Animals fed an HFD are a well established model of non-insulin-dependent type II diabetes, which exhibit many hallmarks of metabolic dysfunction such as reduced glucose tolerance (Surwit et al., 1988; Fontaine and Davis, 2016). In the whole hypothalamus, the expression of *Gli1* was upregulated in lean mice following an overnight fast. Congruent with this finding, the expression of *Ptch1* exhibited a nonsignificant upregulation in lean, fasted animals. In contrast, we observed no significant transcriptional regulation in the hypothalamus of fasted obese animals compared with fed controls. These data suggest that hedgehog signaling is involved in the physiological response to fasting and may be dysregulated in obese animals. To determine whether this fasting-induced upregulation of *Gli1* is a response generated by the whole hypothalamus or specific nuclei, micropunches were collected from hypothalamic nuclei as well as the cortex. We found that in fasted animals *Gli1* expression was elevated specifically in the VMH, PVN, and, to a lesser extent, the cortex, but not the ARC. These data suggest that increased hedgehog pathway activity in the hypothalamus upon fasting is primarily driven by the VMH and PVN. Future studies will determine the cell types responsible within these nuclei for this increase in *Gli1* expression. Additional work is also required to determine the source of the pathway activation observed in these studies. It is possible that ligand is produced outside of the hypothalamus, therefore, further analysis could potentially reveal the primary source of *Shh* following an overnight fast.

Taken as a whole, these data show that neurons of the hypothalamus express both *Shh* and members of its signaling pathway that are required for signal transduction, and that activity of this pathway is upregulated in response to fasting in discrete hypothalamic nuclei. Given our *in situ* data identifying astrocytes as being highly positive for *Gli1* and *Ptch1* in the hypothalamus, it is possible that astrocytes or other support cells are primarily responsible for this fasting-induced upregulation of hedgehog signaling. We have previously shown in primary hypothalamic cultures, consisting of both neurons and glia, that modulation of the hedgehog pathway alters the electrophysiological response to melanin-concentrating hormone (Bansal et al., 2019). Interestingly, it has also been demonstrated in primary cortical cultures that the presence of astrocytes alters the response of neurons to agonism of the hedgehog pathway (Ugbode et al., 2017). Furthermore, astrocyte-specific inhibition of hedgehog signaling *in vivo* was shown to disrupt early postnatal organization and remodeling of cortical synapses resulting in increased neuronal excitability (Hill et al., 2019). Together, these findings suggest novel potential roles for hedgehog signaling outside of its roles as a classical developmental morphogen or in stem cell niche regulation.

The data presented here on the expression and transcriptional regulation of the hedgehog pathway in the adult mouse hypothalamus lays the foundation for future mechanistic studies to determine its role in the proper functioning of the hypothalamus. Given that mammalian hedgehog signaling is coordinated by primary cilia, our future studies will focus on how hypothalamic hedgehog expression may contribute to the obesity phenotype seen in ciliopathies such as Bardet–Biedl syndrome and Alström syndrome (Marshall et al., 2011; Forsythe et al., 2018). Interestingly, certain ciliopathy clinical features such as skeletal and external genitalia abnormalities are also observed in patients with genetic defects in the hedgehog pathway (Umehara et al., 2000; Gao et al., 2001; Hellemans et al., 2003; Mujahid et al., 2018; Khan et al., 2019). Therefore, it would be of interest to determine whether hedgehog signaling is dysregulated in the hypothalamus of animal ciliopathy models. Further mechanistic studies are needed to determine whether hedgehog signaling modulates neuronal activity critical for the physiological response to fasting and whether genetic modulation of the hedgehog pathway in the hypothalamus alters feeding behavior. In conclusion, elucidating the involvement of this developmentally important signaling pathway in feeding behavior and body composition is an exciting new avenue of the hedgehog pathway to explore. Greater understanding of the hedgehog pathway in adult energy homeostasis may also reveal common themes for this pathway in regulation of other behaviors.

## Synthesis

Reviewing Editor: Matthew Grubb, King’s College London Decisions are customarily a result of the Reviewing Editor and the peer reviewers coming together and discussing their recommendations until a consensus is reached. When revisions are invited, a fact-based synthesis statement explaining their decision and outlining what is needed to prepare a revision will be listed below. The following reviewer(s) agreed to reveal their identity: Frances Garcia-Gonzalo, Clemens Kiecker. Note: If this manuscript was transferred from JNeurosci and a decision was made to accept the manuscript without peer review, a brief statement to this effect will instead be what is listed below.

After consultation, both Reviewers agree that the manuscript would be suitable for publication as it is. However, they wanted to give you the option of re-working the text to de-emphasise the immediate relevance for primary cilia, as suggested by Reviewer 2 (see comments below).

If you would like to take that option, please go ahead and submit a revised manuscript, which I will then check without sending out for further review. Otherwise please simply respond through the system with a notification that you’d rather leave the manuscript as it is.

Reviewer 1:

In this manuscript, Antonellis et al. show that Hedgehog (Hh) pathway mRNAs are robustly expressed in hypothalamic feeding control centers like the arcuate (ARC) and periventricular (PVN) nuclei (Figures 1-3), and that the hypothalamic Hh pathway is activated by fasting in control, but not obese, mice (Figure 4).

Overall, I think the article is well written, the data are of good quality, and the findings have important implications. These implications include: (i) evidence that Hh signaling plays an important role in differentiated neurons and glia, in addition to its previously described roles in developmental and adult neurogenesis; and (ii) evidence that fasting-induced hypothalamic Hh signaling is disrupted in high fat diet-fed obese mice, which may help explain the clinical manifestations of some ciliopathies.

A few months ago, I reviewed this paper for another journal. All the points I raised there, regarding inclusion of Ptch1 data in several figures, have already been satisfactorily addressed by the authors in this new version for eNeuro. Based on this, and on the aforementioned quality and relevance of the data, I recommend acceptance of this manuscript without further delay.

Reviewer 2:

This is an interesting extension of your previous work (Bansal et al. 2019) that demonstrates that hedgehog pathway genes are expressed in specific neurons in feeding centres in the mouse hypothalamus and that their expression is regulated by nutritional state/fasting. However, I am not sure about the organisation of the manuscript: your previous work (and that of others) has of course linked primary cilia with hedgehog signalling in vertebrates, yet, cilia are not directly targeted or shown in the present study. Nevertheless, Abstract, Introduction and Discussion all start with passages on primary cilia, drawing the reader’s focus towards this topic. I recommend that you focus on hedgehog signalling, the hypothalamus and feeding and mention cilia later in the Introduction and Discussion in order to avoid a skewed emphasis in the manuscript.
